# Supplementary material for: Multi‐dataset identification of innovative feature genes and molecular mechanisms in keratoconus
Source: J Cell Mol Med. 2024 Sep 19;28(18):e70079. doi: 10.1111/jcmm.70079 (PMC11412914; doi:10.1111/jcmm.70079)
Supplement: Supplementary file 2 — Material S2. [file JCMM-28-e70079-s002.zip › 7.GSEA analysis/4.MYRF/KEGG_OOCYTE_MEIOSIS.html]

Details for gene set KEGG\_OOCYTE\_MEIOSIS[GSEA]

|  || Dataset | MYRF.group.cls#h\_versus\_l |
| Phenotype | group.cls#h\_versus\_l |
| Upregulated in class | h |
| GeneSet | KEGG\_OOCYTE\_MEIOSIS |
| Enrichment Score (ES) | 0.18340456 |
| Normalized Enrichment Score (NES) | 0.92097515 |
| Nominal p-value | 0.5728543 |
| FDR q-value | 1.0 |
| FWER p-Value | 1.0 |
Table: GSEA Results Summary

  

Fig 1: Enrichment plot: KEGG\_OOCYTE\_MEIOSIS      
 Profile of the Running ES Score & Positions of GeneSet Members on the Rank Ordered List

  

| SYMBOL | TITLE | RANK IN GENE LIST | RANK METRIC SCORE | RUNNING ES | CORE ENRICHMENT || 1 | CAMK2B | na | 283 | 0.659 | 0.0457 | Yes |
| 2 | ADCY1 | na | 1675 | 0.385 | 0.0370 | Yes |
| 3 | AR | na | 1716 | 0.381 | 0.0671 | Yes |
| 4 | PPP3R2 | na | 2013 | 0.356 | 0.0876 | Yes |
| 5 | CCNE2 | na | 2609 | 0.317 | 0.0964 | Yes |
| 6 | ADCY2 | na | 2688 | 0.312 | 0.1197 | Yes |
| 7 | CHP2 | na | 3253 | 0.283 | 0.1266 | Yes |
| 8 | CAMK2A | na | 4292 | 0.245 | 0.1166 | Yes |
| 9 | ADCY5 | na | 4506 | 0.238 | 0.1299 | Yes |
| 10 | FBXO43 | na | 4654 | 0.233 | 0.1447 | Yes |
| 11 | CPEB1 | na | 4824 | 0.228 | 0.1585 | Yes |
| 12 | RPS6KA2 | na | 4975 | 0.223 | 0.1724 | Yes |
| 13 | CALML6 | na | 5567 | 0.206 | 0.1722 | Yes |
| 14 | IGF1R | na | 7135 | 0.160 | 0.1400 | Yes |
| 15 | ITPR2 | na | 7235 | 0.158 | 0.1500 | Yes |
| 16 | CALML5 | na | 7292 | 0.156 | 0.1612 | Yes |
| 17 | ANAPC1 | na | 7351 | 0.154 | 0.1721 | Yes |
| 18 | SMC1A | na | 8302 | 0.131 | 0.1555 | Yes |
| 19 | MAPK12 | na | 8572 | 0.125 | 0.1579 | Yes |
| 20 | RPS6KA6 | na | 8598 | 0.125 | 0.1674 | Yes |
| 21 | ADCY7 | na | 8605 | 0.125 | 0.1774 | Yes |
| 22 | IGF1 | na | 8743 | 0.121 | 0.1834 | Yes |
| 23 | ESPL1 | na | 9710 | 0.101 | 0.1638 | No |
| 24 | ADCY4 | na | 9919 | 0.097 | 0.1658 | No |
| 25 | CAMK2G | na | 10986 | 0.078 | 0.1414 | No |
| 26 | CCNE1 | na | 11231 | 0.075 | 0.1404 | No |
| 27 | CDK1 | na | 11292 | 0.073 | 0.1447 | No |
| 28 | PRKX | na | 11663 | 0.068 | 0.1396 | No |
| 29 | PPP2R5A | na | 11827 | 0.066 | 0.1403 | No |
| 30 | CALM2 | na | 11947 | 0.064 | 0.1421 | No |
| 31 | RPS6KA3 | na | 12372 | 0.058 | 0.1346 | No |
| 32 | CDC23 | na | 12691 | 0.054 | 0.1299 | No |
| 33 | ADCY6 | na | 13405 | 0.045 | 0.1130 | No |
| 34 | YWHAB | na | 13423 | 0.045 | 0.1161 | No |
| 35 | ANAPC7 | na | 13808 | 0.040 | 0.1083 | No |
| 36 | MAPK3 | na | 14382 | 0.034 | 0.0945 | No |
| 37 | PGR | na | 14733 | 0.030 | 0.0869 | No |
| 38 | RBX1 | na | 14789 | 0.030 | 0.0877 | No |
| 39 | SMC1B | na | 15002 | 0.028 | 0.0839 | No |
| 40 | PPP2R1B | na | 15086 | 0.027 | 0.0837 | No |
| 41 | ANAPC13 | na | 15453 | 0.023 | 0.0750 | No |
| 42 | YWHAZ | na | 16350 | 0.014 | 0.0503 | No |
| 43 | FBXW11 | na | 16395 | 0.014 | 0.0502 | No |
| 44 | ANAPC4 | na | 16590 | 0.012 | 0.0456 | No |
| 45 | YWHAE | na | 16676 | 0.011 | 0.0440 | No |
| 46 | BTRC | na | 16745 | 0.011 | 0.0429 | No |
| 47 | CALML3 | na | 17129 | 0.008 | 0.0325 | No |
| 48 | SKP1 | na | 17295 | 0.006 | 0.0282 | No |
| 49 | ANAPC11 | na | 17415 | 0.005 | 0.0252 | No |
| 50 | ADCY9 | na | 17667 | 0.003 | 0.0181 | No |
| 51 | CALM1 | na | 17693 | 0.002 | 0.0176 | No |
| 52 | PPP3R1 | na | 17799 | 0.001 | 0.0147 | No |
| 53 | SLK | na | 17871 | 0.001 | 0.0127 | No |
| 54 | ITPR3 | na | 17996 | -0.000 | 0.0091 | No |
| 55 | PPP1CB | na | 18335 | -0.004 | -0.0003 | No |
| 56 | PTTG2 | na | 18336 | -0.004 | -0.0000 | No |
| 57 | PPP1CA | na | 18340 | -0.004 | 0.0002 | No |
| 58 | ANAPC5 | na | 18440 | -0.005 | -0.0023 | No |
| 59 | PPP2R5D | na | 18454 | -0.005 | -0.0023 | No |
| 60 | ITPR1 | na | 18799 | -0.008 | -0.0115 | No |
| 61 | PTTG1 | na | 19038 | -0.011 | -0.0175 | No |
| 62 | CHP1 | na | 19165 | -0.012 | -0.0201 | No |
| 63 | CDC16 | na | 19192 | -0.013 | -0.0199 | No |
| 64 | PRKACA | na | 19584 | -0.017 | -0.0298 | No |
| 65 | CDC27 | na | 19757 | -0.019 | -0.0332 | No |
| 66 | PPP2R5C | na | 19773 | -0.019 | -0.0321 | No |
| 67 | PPP2R5E | na | 19812 | -0.020 | -0.0316 | No |
| 68 | STAG3 | na | 19930 | -0.021 | -0.0332 | No |
| 69 | MOS | na | 20086 | -0.023 | -0.0358 | No |
| 70 | ADCY8 | na | 20359 | -0.026 | -0.0416 | No |
| 71 | MAPK1 | na | 20406 | -0.027 | -0.0407 | No |
| 72 | YWHAG | na | 20695 | -0.030 | -0.0466 | No |
| 73 | CUL1 | na | 21468 | -0.039 | -0.0657 | No |
| 74 | MAD2L1 | na | 22188 | -0.048 | -0.0825 | No |
| 75 | PPP3CA | na | 22219 | -0.049 | -0.0794 | No |
| 76 | AURKA | na | 22641 | -0.054 | -0.0872 | No |
| 77 | PPP2R1A | na | 22646 | -0.054 | -0.0828 | No |
| 78 | PPP3CB | na | 22763 | -0.056 | -0.0816 | No |
| 79 | YWHAQ | na | 22775 | -0.056 | -0.0773 | No |
| 80 | PPP1CC | na | 22860 | -0.057 | -0.0751 | No |
| 81 | PLCZ1 | na | 22984 | -0.059 | -0.0738 | No |
| 82 | CALM3 | na | 23602 | -0.069 | -0.0859 | No |
| 83 | CDC26 | na | 23958 | -0.075 | -0.0901 | No |
| 84 | ANAPC10 | na | 24062 | -0.077 | -0.0867 | No |
| 85 | ANAPC2 | na | 24204 | -0.079 | -0.0843 | No |
| 86 | PPP2R5B | na | 24593 | -0.086 | -0.0885 | No |
| 87 | REC8 | na | 24766 | -0.089 | -0.0862 | No |
| 88 | CCNB2 | na | 25069 | -0.094 | -0.0872 | No |
| 89 | CDC20 | na | 25083 | -0.095 | -0.0798 | No |
| 90 | SPDYA | na | 25480 | -0.102 | -0.0829 | No |
| 91 | PPP2CB | na | 25840 | -0.109 | -0.0843 | No |
| 92 | SMC3 | na | 25918 | -0.111 | -0.0775 | No |
| 93 | FBXO5 | na | 26037 | -0.114 | -0.0716 | No |
| 94 | BUB1 | na | 26277 | -0.119 | -0.0688 | No |
| 95 | CCNB1 | na | 26279 | -0.119 | -0.0591 | No |
| 96 | PPP2CA | na | 26826 | -0.131 | -0.0641 | No |
| 97 | PKMYT1 | na | 26932 | -0.134 | -0.0562 | No |
| 98 | ADCY3 | na | 26955 | -0.134 | -0.0459 | No |
| 99 | MAD2L2 | na | 27045 | -0.136 | -0.0373 | No |
| 100 | SPDYC | na | 27254 | -0.141 | -0.0318 | No |
| 101 | PPP3CC | na | 28351 | -0.171 | -0.0494 | No |
| 102 | RPS6KA1 | na | 29168 | -0.196 | -0.0570 | No |
| 103 | CDK2 | na | 29418 | -0.203 | -0.0475 | No |
| 104 | MAP2K1 | na | 30150 | -0.229 | -0.0499 | No |
| 105 | INS | na | 30231 | -0.232 | -0.0333 | No |
| 106 | PRKACG | na | 31385 | -0.285 | -0.0433 | No |
| 107 | YWHAH | na | 31453 | -0.288 | -0.0216 | No |
| 108 | CAMK2D | na | 31910 | -0.312 | -0.0093 | No |
| 109 | CDC25C | na | 31922 | -0.313 | 0.0160 | No |
| 110 | PLK1 | na | 32621 | -0.359 | 0.0253 | No |
| 111 | PRKACB | na | 33438 | -0.435 | 0.0373 | No |
Table: GSEA details [plain text format]

  

Fig 2: KEGG\_OOCYTE\_MEIOSIS      
 Blue-Pink O' Gram in the Space of the Analyzed GeneSet

  

Fig 3: KEGG\_OOCYTE\_MEIOSIS: Random ES distribution      
 Gene set null distribution of ES for **KEGG\_OOCYTE\_MEIOSIS**
